# Medication‐induced mood disorders following epidural steroid injections in patients with pain: A case series

**DOI:** 10.1002/ccr3.7415

**Published:** 2023-05-25

**Authors:** Christian T. Vangeison, Joseph P. Roys, Marissa N. Catalanotto, Wyatt I. Kupperman, Alexander R. Kovacs, Dan Sebastian Dîrzu, Emanuel N. Husu

**Affiliations:** ^1^ H. Ben Taub Department of Physical Medicine & Rehabilitation Baylor College of Medicine Houston Texas USA; ^2^ University of Buffalo Jacobs School of Medicine Buffalo New York USA; ^3^ Anesthesia and Intensive Care Department Emergency County Hospital Cluj‐Napoca Cluj Napoca Romania; ^4^ Department of Clinical Sciences, Chicago Medical School Rosalind Franklin University of Medicine and Science North Chicago Illinois USA

**Keywords:** case report, epidural steroid injection induced mood disorder, medication‐induced mood disorder, substance‐induced mood disorder

## Abstract

**Key Clinical Message:**

Medication‐induced mood disorders following epidural steroid injections are possible therefore should be disclosed to the patient.

**Abstract:**

Medication‐induced mood disorders have been rarely reported following epidural steroid injections (ESI). This case series presents three patients who met the Diagnostic and Statistical Manual of Mental Disorders, Fifth Edition (DSM‐5) criteria for substance/medication‐induced mood disorder after an ESI. In considering a candidacy for ESI, the rare but significant, side effects of psychiatric side effects should be disclosed to patients.

## 1 INTRODUCTION

Depression and anxiety disorders are complex psychiatric illnesses that can occur with or without an inciting event. It is well known mood disorders can affect many facets of a person's life, notably their perception of pain. Conversely, it is also established that pain, whether acute, subacute, or chronic, may also precipitate mood disturbances. The symptoms of mood disorders can include hypomania/mania, depression, anxiety, suicidal ideation, delirium, and psychosis.

Chen et al. reported a case of mania following epidural steroid injections (ESI) in a patient without a previous psychiatric history.^1^ Furthermore, a case series observed up to 52.5% of lifetime systemic glucocorticoid users experienced one or more mood‐related symptoms, with 11.3% experiencing depression.^2^ Kenna et al. in a review of psychiatric complications with corticosteroid use found that the mean prednisone‐dose equivalent was 63.6 mg/day, supporting previous studies that higher doses are more likely to have side effects.^3^ However, to date, there are few published cases of ESI inducing depression in a patient. So far, there have also been limited cases published of an ESI inducing the Diagnostic and Statistical Manual of Mental Disorders, Fifth Edition (DSM‐5) criteria for substance/medication‐induced depressive disorder.

There are five criteria to meet the DSM‐5 diagnosis for a substance/medication‐induced depressive disorder. These are (1) persistent disturbance in mood characterized by depressed mood or anhedonia, (2) symptoms of depression or anhedonia develop soon after suspected exposure to medication/substance or the involved medication/substance is known to be capable of producing such symptoms, (3) the disturbance is not better explained by another depressive disorder or depressive symptoms resolve after removal of medication/substance, (4) depressive disturbance is not occurring during a time of delirium, (5) the depressive symptoms are causing significant distress in a person's life‐limiting his/her ability to function.^4^ There is no consensus on the treatment of substance/medication‐induced mood disorders. This is attributed to the uniqueness of each individual's depressive episode and the possibility of another superimposed psychiatric disorder(s). This case series will outline three patients who experienced significant mood symptoms following an ESI. Although each case differs in presentation, all highlight the need for physicians to be keenly aware of developing mood symptoms before and after an ESI. This paper calls for further mood symptom screening and monitoring for patients and a swift plan of action if and when mood changes are detected.”

## 2 CASE HISTORY

Case one is a 32‐year‐old African American female with no past medical history who presented to the pain management clinic with complaints of pain after a motor vehicle collision occurring 1 month prior to presentation. She reported the pain as being 8/10, radiating to her left buttock and mid‐posterior thigh. Two weeks before the visit, she was prescribed an oral methylprednisolone dose pack for 6 days, which relieved her pain without observed side effects. Her examination was significant for bilateral L5 4/5 weakness with 1/2 sensation to light touch. Magnetic resonance imaging (MRI) of the lumbar spine revealed type one modic changes of the inferior and superior plates of L5 and S1 with a large right central and left central disc protrusion resulting in contact of the bilateral descending S1 nerve roots. She then underwent bilateral L5–S1 transforaminal ESIs with an injectate of 1 mL of 40 mg/mL triamcinolone and 1 mL of 0.25% bupivacaine. She reported nausea and vomiting the day of the injection and insomnia that lasted 6 days. Seven weeks post‐injection, she had mood changes, swelling in her face and hands, polymenorrhagia, and weight gain and was referred to her endocrinology by per primary care provider. Ten weeks post‐injection, she presented to the endocrinology clinic by referral from her primary care provider due persistent weight gain and was subsequently diagnosed with iatrogenic steroid‐induced Cushing syndrome after being found to have an elevated cortisol level. She was referred by her primary care provider to psychiatry 4 months post‐ESI. She was started on sertraline 25 mg titrated up to 50 mg and prazosin 1 mg at bedtime, after having been diagnosed with post‐traumatic stress disorder, severe major depressive disorder (MDD), and panic disorder. The patient was seen by the pain psychology clinic for pain coping strategies. When re‐evaluated by a pain management physician in clinic nearly three and a half years later, she was off sertraline or prazosin without further depression.

Case two is a 58‐year‐old caucasian male with a past medical history significant for hypertension who was evaluated by pain medicine for chronic low back pain associated with radicular pain down the left leg. It was later discovered that the patient had a remote history of MDD over 20 years prior treated with venlafaxine and bupropion. His examination was significant for 4+/5 strength in left L4 distribution with left L4 1/4 reflexes. MRI of the lumbar spine revealed lateral recess and foraminal stenosis at L4–L5 and L5–S1 with probable compression of the traversing left L5 and exiting left L4 nerve roots. He underwent a left L4–L5 transforaminal ESI under fluoroscopic guidance consisting of a mixture of 1 mL of 0.5% bupivacaine and 40 mg of triamcinolone. At one‐week post‐injection, the patient reported his mood had severely worsened, similar to a depressive episode he had suffered many years prior. He noted the onset occurred 2 days post‐ESI without any pre‐procedure depressive symptoms. The patient experienced anhedonia and suicidal ideation without a plan. He promptly resumed his former depression treatment regimen of venlafaxine 75 mg daily and bupropion 100 mg daily under the supervision of his primary care physician (PCP). At 3 weeks post‐ESI, the patient reported an 80% improvement in his pain, although his mood remained depressed. At that time, his PCP titrated his bupropion up to 200 mg daily and referred him to a psychologist and psychiatrist. His depressive symptoms fully resolved 3 months post‐ESI.

Case three is a 56‐year‐old Hispanic male with a past medical history of depression and anxiety presenting to pain medicine for evaluation of 3 days of mid‐thoracic back pain. He rated the constant, burning pain as 10/10 and stated that it was exacerbated with any movement, specifically back extension. Physical examination was notable for tenderness to palpation in the left thoracic paraspinals and left thoracolumbar facet joint loading. The examination was largely limited due to pain but was without signs of weakness or neurological compromise. MRI of the thoracic spine revealed multilevel discogenic and facet changes throughout the thoracic spine. He underwent a successful T12–L1 interlaminar steroid injection after his insurance denied left‐sided thoracic medial branch blocks. Interlaminar injectate included triamcinolone 80 mg with 3 cc of 1% lidocaine & 5 cc of normal saline. Two days after the injection, he reported new‐onset insomnia. At his follow‐up visit, when asked about his mood, he stated “I am scared” referring to his anxiety, with a concordant generalized anxiety disorder‐7 (GAD‐7) score of 15. To curb his insomnia, he was prescribed a four‐week course of trazodone. It was recommended by the pain management team that he follow‐up with his psychiatrist and other mental health providers. At the three‐month follow‐up, he reported persistent sleep and appetite disturbances with associated weight loss and anhedonia with an inability to return to work due to his mood and pain. By the 5‐month follow‐up, all symptoms had resolved with a GAD‐7 score of 4. His psychiatrist had prescribed citalopram 5 mg daily for the previous 2 months.

## 3 DISCUSSION

ESI‐induced depression may be an overlooked side effect of steroid injections. Mood symptoms may occur more frequently than originally thought as subclinical symptoms may go unreported by many chronic pain patients. Following up on a detailed post‐injection psychiatric history is a challenging endeavor. Many patients have a complex psychiatric history characterized by multiple comorbid psychiatric syndromes that make it difficult to anchor new mood symptoms to an exogenous substance. Kim et al. studied patients with and without depression that had been diagnosed with cervical spondylosis and cervical radicular pain finding a statistically significant difference between groups for reported pain improvement. The non‐depressed group had greater pain improvement documented at all follow‐up checkpoints.^5^

Patients who experience pain are predisposed to developing mood disorders. While an in‐depth discussion of the links between pain and depression goes beyond the scope of this paper, the association is likely due to overlapping neural pathways and neurotransmitters, among other similarities that lead to maladaptive neuroplasticity.^6^ Special consideration may be necessary before the administration of ESIs in patients being prescribed long‐term steroids. In patients, such as those with chronic obstructive pulmonary disorder or an autoimmune disorder, a dose‐dependent correlation between steroid use and psychiatric symptoms has been observed.^5^ Patients who are receiving long‐term systemic steroids are at an increased risk of psychiatric complications. The administration of an ESI may further increase their risk. For this reason, additional screening should be considered to rule out a history of long‐term steroid use.

If signs and symptoms of depression are recognized, a prompt referral to a psychiatrist should be made. If an immediate psychiatry appointment cannot be scheduled, patients should follow‐up with their PCP. If patients report depression with suicidal ideation, they should be urgently directed to the nearest emergency room or crisis center.

In case one, during the initial pain management screening, the patient did not endorse depression or anxiety in the clinic screening. When evaluated by psychiatry, steroid‐induced depression was not on the differential diagnosis and the recent ESI was not mentioned in the note. Those with Cushing syndrome can have several neurophysiologic symptoms including depression (37%–86%), anxiety (80%), panic attacks (30%), irritability (8%), insomnia, memory loss, and emotional lability, while mild paranoia and mania are less common.^6^, ^7^, ^8^, ^9^, ^10^ The psychiatric manifestations observed in Cushing can be attributed to excess cortisol,^6^, ^7^, ^8^ with many patients having psychiatric symptom(s) as a part of the initial presenting symptom(s).^9^, ^10^ Patients who experience severe depression need to be monitored closely for signs of suicidality.^6^, ^7^

Concerning cases two and three, it is not clear if the patients were screened for a history of mood disorders at their initial evaluations. Both patients' mental health histories were recorded based on intake forms. However, it does not appear that further consideration of previous mood disorders was taken into consideration with ESI planning. It is important to note that his symptoms were not immediately linked to the ESI. In the third case, symptoms were initially masked by the confounding acuity of his pain exacerbation and new‐onset insomnia after the ESI. It was not until the three‐month follow‐up that symptoms seemed to have developed into more easily recognized mood symptomatology including anhedonia, appetite changes, weight loss, and continued sleep disturbance. The latter two cases highlight the value of a pain management physician using a patient health questionnaire‐9 (PHQ‐9), GAD‐7, or a similar screening tool in the initial intake before visits, especially in cases where procedures may be indicated. More so, these screening tools can be used to assess mood‐related side effects in the immediate and intermediate post‐procedure time frames.

The authors recognize that pain may precipitate mood dysregulation and may be a cofounding variable in the cases discussed. A definitive causation cannot be attributed entirely due to an ESI, and the association between pain and mood disturbance is acknowledged. Based upon symptom onset and presentation, an ESI may have been one of the precipitants of mood dysregulation in the three cases discussed. Although definitive causation cannot be attributed entirely due to this variable, it appears likely from the symptom onset and presentation that an ESI(s) was the most likely precipitant of mood dysregulation in the three cases discussed. In considering candidacy for ESIs, the rare, but significant side effects of depression or other psychiatric manifestations should be discussed with patients at a minimum during the consent process. This potentially life‐altering side effect should especially be disclosed as part of the informed consent process to those who have had or are currently suffering from depression. A pain management physician should consider pre‐screening patients for risk factors as well as have a management plan that includes prompt referral to psychiatry if psychiatric symptoms are reported by the patient.

## AUTHOR CONTRIBUTIONS

**Christian T Vangeison:** Conceptualization; methodology; writing – original draft; writing – review and editing. **Joseph p Roys:** Conceptualization; methodology; writing – original draft; writing – review and editing. **Marissa N Catalanotto:** Conceptualization; methodology; writing – review and editing. **Wyatt I Kupperman:** Methodology; supervision; writing – review and editing. **Alexander R Kovacs:** Conceptualization; methodology; writing – original draft; writing – review and editing. **Dan Sebastian Dîrzu:** Supervision; writing – review and editing. **Emanuel N Husu:** Conceptualization; project administration; supervision; writing – review and editing.

## FUNDING INFORMATION

The authors have no sources of funding or competing interests to declare for this manuscript.

## CONFLICT OF INTEREST STATEMENT

The authors declare no conflicts of interest.

## CONSENT STATEMENT

Written informed consent was obtained from the patient to publish this report in accordance with the journal's patient consent policy.

## Data Availability

The data that support the findings of this study are available on request from the corresponding author, upon reasonable request.

## References

[1] Fardet L , Flahault A , Kettaneh A , et al. Corticosteroid‐induced clinical adverse events: frequency, risk factors and patient's opinion. Br J Dermatol. 2007;157(1):142‐148. doi:10.1111/j.1365-2133.2007.07950.x 17501951

[2] Kenna HA , Poon AW , de los Angeles CP , Koran LM . Psychiatric complications of treatment with corticosteroids: review with case report. Psychiatry Clin Neurosci. 2011;65(6):549‐560. doi:10.1111/j.1440-1819.2011.02260.x 22003987

[3] Revadigar N , Gupta V . Substance Induced Mood Disorders. StatPearls Publishing; 2021. 32310347

[4] Kim EJ , Chotai S , Schneider BJ , Sivaganesan A , McGirt MJ , Devin CJ . Effect of depression on patient‐reported outcomes following cervical epidural steroid injection for degenerative spine disease. Pain Med. 2018;19(12):2371‐2376. doi:10.1093/pm/pny196 30357417

[5] Plotz CM , Knowlton AI , Ragan C . The natural history of Cushing's syndrome. Am J Med. 1952;13(5):597‐614. doi:10.1016/0002-9343(52)90027-2 12996538

[6] Haskett RF . Diagnostic categorization of psychiatric disturbance in Cushing's syndrome. Am J Psychiatry. 1985;142(8):911‐916. doi:10.1176/ajp.142.8.911 2992298

[7] Kelly WF . Psychiatric aspects of Cushing's syndrome. QJM. 1996;89(7):543‐551. doi:10.1093/qjmed/89.7.543 8759496

[8] Loosen PT , Chambliss B , DeBold CR , Shelton R , Orth DN . Psychiatric phenomenology in Cushing's disease. Pharmacopsychiatry. 1992;25(4):192‐198. doi:10.1055/s-2007-1014405 1528959

[9] Starkman MN . Neuropsychiatric findings in Cushing syndrome and exogenous glucocorticoid administration. Endocrinol Metab Clin North Am. 2013;42(3):477‐488. doi:10.1016/j.ecl.2013.05.010 24011881

[10] Dorn LD , Burgess ES , Dubbert B , et al. Psychopathology in patients with endogenous Cushing's syndrome: 'atypical' or melancholic features. Clin Endocrinol (Oxf). 1995;43(4):433‐442. doi:10.1111/j.1365-2265.1995.tb02614.x 7586617

